# *Polygonatum sibiricum* Polysaccharides Attenuate Lipopoly-Saccharide-Induced Septic Liver Injury by Suppression of Pyroptosis via NLRP3/GSDMD Signals

**DOI:** 10.3390/molecules27185999

**Published:** 2022-09-15

**Authors:** Linxia Xiao, Liang Qi, Guozhe Zhang, Hongxia Liu, Yaqin Gu, Lihu Zhang, Mingguang Zhang, Hongyan Wu

**Affiliations:** 1 School of Pharmacology, Jiangsu Vocational College of Medicine, Yancheng 224005, China; 2 Institute of Biomedical Technology, Jiangsu Vocational College of Medicine, Yancheng 224005, China

**Keywords:** sepsis, acute liver injury, *Polygonatum sibiricum* polysaccharides, pyroptosis, GSDMD, NLRP3

## Abstract

Sepsis is a systemic inflammatory response syndrome with high mortality. Acute liver injury is an independent predictor for poor prognosis in septic patients. *Polygonatum sibiricum* polysaccharides (PSP) have been reported to possess anti-inflammatory and hepatoprotective activities. To evaluate the effects of PSP on septic liver injury and demonstrate the potential molecular mechanisms, the septic acute liver injury (SALI) model was established in BALB/c mice via intraperitoneal injection of lipopolysaccharide (LPS). We found that PSP treatment could remarkably reduce the 48 h mortality rate of septic mice; alleviate liver histopathologic damage; lower the activity of neutrophil infiltration marker MPO in liver tissue; and decrease the levels of liver function indexes AST, ALT, ALP, and TBIL, inflammatory cytokines TNFα and IL-6, and pyroptosis-related inflammatory cytokines IL-18 and IL-1β in serum. TUNEL staining and detecting GSDMD-NT protein expression level in liver tissue revealed that PSP could restrain excessive pyroptosis. In addition, PSP treatment reversed the upregulations of mRNA expression levels of the NLRP3/GSDMD signals in the liver. Our results indicated the potential protective role of PSP against SALI by inhibiting pyroptosis via NLRP3/GSDMD signals.

## 1. Introduction

Sepsis, a life-threatening disease, is caused by the systemic inflammatory response triggered by infection, trauma, burns, or toxin [1]. It is one of the primary causes of death globally, with mortality ranging up to 25–30% [2]. Moreover, the incidence rate of sepsis has been rising worldwide in recent years [3]. Therefore, sepsis imposes a huge burden on public health and the economy around the world [4]. It can further induce multiple organ dysfunction, including heart, brain, liver, lung, and kidney, which results in worsened outcomes [5]. Therefore, as a pivotal organ for maintaining host homeostasis and clearing infectious agents, the liver has significant effects on the pathogenesis of sepsis [6]. Regrettably, sepsis can cause acute liver injury via hemodynamic alterations or inducing insults to hepatocytes [7]. According to the literature, acute liver injury is a crucial factor resulting in a poor clinical outcomes in sepsis [8,9], and ameliorating liver injury is a pivotal component in decreasing septic death [10]. However, there are few therapeutic approaches available for the attenuation of SALI, and its exact pathogenesis is intricate and vague [6,11]. Hence, it is vital to investigate pathophysiological changes and develop more effective therapies for SALI.

In early sepsis, pyroptosis, recognized as a primary defense mechanism of the body, can initiate immunocyte to engulf and kill pathogens and therefore alleviate organ and tissue damage [12]. Pyroptosis, a newfound regulated cell death, is characterized by its caspase-dependent pathway and pore formation by the cleaved GSDMD [13,14]. There are two signaling pathways of pyroptosis, including the caspase-1-dependent canonical pathway and caspase-4/5/11-dependent non-canonical pathway. In the canonical pathway, NLRP3 inflammasome, a multiprotein complex made up of the innate immune sensor molecule NLRP3, adapter protein ASC, and pro-caspase-1, has an integral role in initiating pyroptosis [15]. When cells suffer from microbial infection or damage, the pattern recognition receptor NLRP3 will recognize the stimuli and then form the NLRP3 inflammasome through ASC mediately linked with pro-caspase-1 [16]. The formation of the inflammasome results in the self-cleavage of pro-caspase-1, which produces activated caspase-1. Subsequently, cleaved caspase-1 causes the cleavage of the pro-IL-1β, pro-IL-18 to generate activated IL-1β and IL-18. Meanwhile, activated caspase-1 promotes the cleavage of pro-GSDMD to release the N-terminal of GSDMD (GSDMD-NT), which results in pore formation and ultimately drives pyroptosis. In the non-canonical pathway, caspase-11 (caspase-4/5 in humans) can be triggered to oligomerize by binding to LPS and then activating [17]. Activated caspase-11 further triggers the cleavage of pro-GSDMD to form membrane perforation and the subsequent secretion of inflammatory cytokines IL-1β and IL-18, eventually resulting in pyroptosis [18], whereas excessive pyroptosis of host cells may elicit uncontrolled inflammation and eventually lead to multiple organ failure, including acute liver injury [19]. Therefore, the suppression of pyroptosis might be an effective therapeutic measure for SALI.

*Polygonatum sibiricum* (PS)—a dry rhizome of *Polygonatum cytomema* Hua, *Polygonatum kingianum* Coll. Et Hemsl., or *Polygonatum sibiricum* Red—is an edible traditional Chinese medicine which has been widely used due to its multiple biological activities, including antioxidant, anti-aging, anti-diabetic, neuroprotective, and immunity enhancement activities among others [20,21,22]. The extensive bioactivities have been ascribed to the existence of multiple active ingredients, such as polysaccharides, flavones, steroid saponins, alkaloids, and amino acids [22]. Among the bioactive ingredients, *Polygonatum sibiricum* polysaccharides (PSP) extracted from PS are considered some of the most crucial components [23]. Furthermore, they have the advantage of low toxicity and can be applied to prolonged clinical therapy [24]. Several studies have demonstrated that PSP exert a variety of biological properties, such as antibacterial, antioxidant, antiatherosclerosis, immunomodulatory, and hepatoprotective properties [25,26]. Additionally, PSP can exert a protective effect on various cells and tissues by inhibiting the inflammatory response. Wang et al. have reported that PSP protects retinal pigment epithelium cells ARPE-19 from high glucose-induced damage by regulating oxidative stress reaction, inflammatory response and apoptosis index [27]. In an LPS-induced depression model, PSP alleviate brain tissue injury through suppressing the signaling pathway of oxidative stress-calpain-1-NLRP3 inflammasome [28]. In addition, PSP have been reported to protect kidney tissues from gentamicin-induced acute damage through inhibiting inflammatory response triggered by p38 MAPK/ATF2 signaling pathway [29]. However, whether PSP exert protective effect on SALI has not been reported. In this paper, we have investigated the effects of PSP on SALI via assessing the changes in hepatic histopathology, detecting the level of biomarker of liver injury and neutrophil infiltration as well as inflammatory cytokine, and evaluating the mRNA and protein level of genes related to NLRP3-inflammasome-mediated pyroptosis.

## 2. Results

### 2.1. PSP Increased Survival Rates of Septic Mice

**Figure 1:**
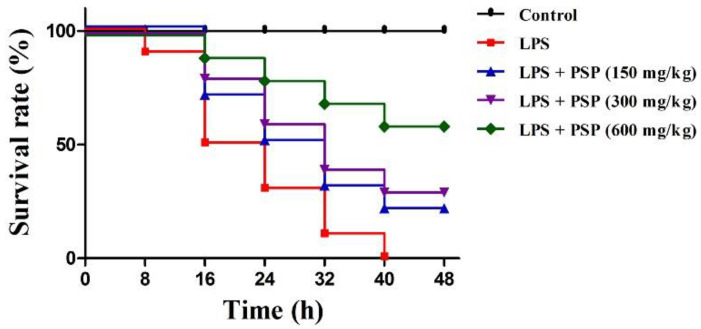
Effects of PSP on the survival of LPS-induced septic mice (*n* = 10).

We first explored whether PSP affords a survival advantage to septic mice. As described in Figure 1, was no mice died in the control group during the experiment, whereas mice in the LPS group began to die at 8 h, and the mortality rate increased to 100% at 48 h. Compared to the LPS group, the 48 h survival rate was significantly improved by PSP gavage in a dose-dependent manner through log-rank test analysis.

### 2.2. PSP Alleviated Acute Liver Injury of Septic Mice

**Figure 2:**
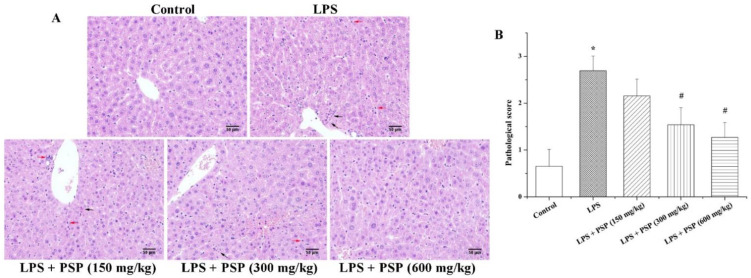
Effects of PSP on liver injury of LPS-induced septic mice. (**A**) HE staining images (red arrow indicates inflammatory cell infiltration; black arrow for cell spotty necrosis and nuclear pyknosis fragmentation, *n* = 6, 400×); (**B**) pathological scores. Data are expressed as mean ± SEM. * *p* < 0.05 versus control group, # *p* < 0.05 versus LPS group.

To investigate the effects of PSP on acute liver injury in septic mice, we first assessed the hepatic histopathological changes of each group by HE staining, and the degree of liver injury was scored based on previous reports [30,31] (Figure 2). The hepatic morphology and architecture were normal in the control group. Compared to the control group, the LPS group showed obvious inflammatory cell infiltration, cell spotty necrosis, and nuclear pyknosis fragmentation, demonstrating severe septic liver injury. Interestingly, treatment with PSP remarkably alleviated the injury induced by LPS.

**Figure 3:**
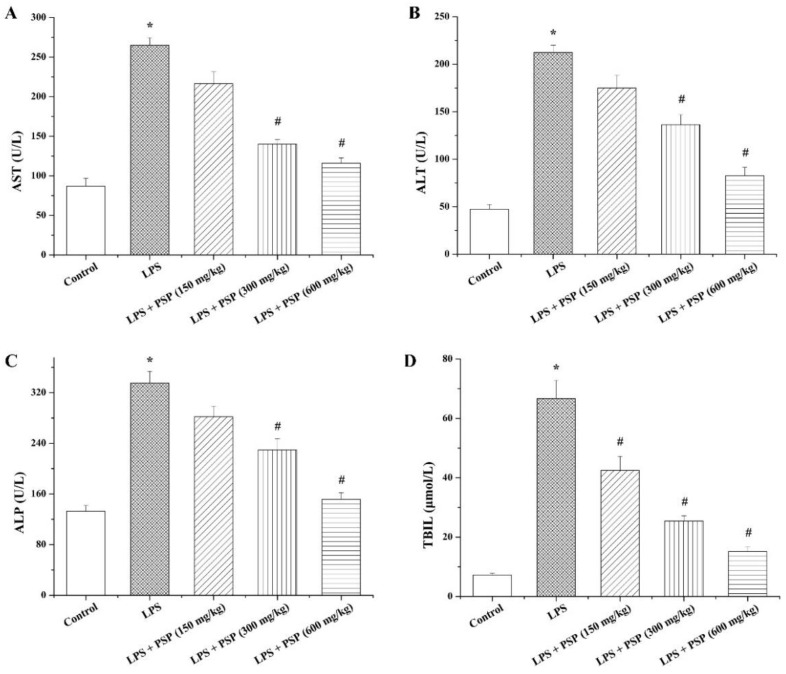
Effects of PSP on liver function indexes in serum of LPS-induced septic mice (*n* = 6). (**A**) Serum AST; (**B**) serum ALT; (**C**) serum ALP; (**D**) serum TBIL. Data are expressed as mean ± SEM. * *p* < 0.05 versus control group, # *p* < 0.05 versus LPS group.

To further estimate the status of liver injury, the levels of liver function indexes AST, ALT, ALP, and TBIL in serum were determined (Figure 3). In comparison with the control group, the levels of AST, ALT, ALP, and TBIL in the LPS group were markedly elevated, and the increased degrees of those indexes were effectively attenuated by PSP treatment in a dose-dependent manner.

### 2.3. PSP Suppressed MPO Activity and Inflammatory Cytokine Secretion of Septic Mice

**Figure 4:**
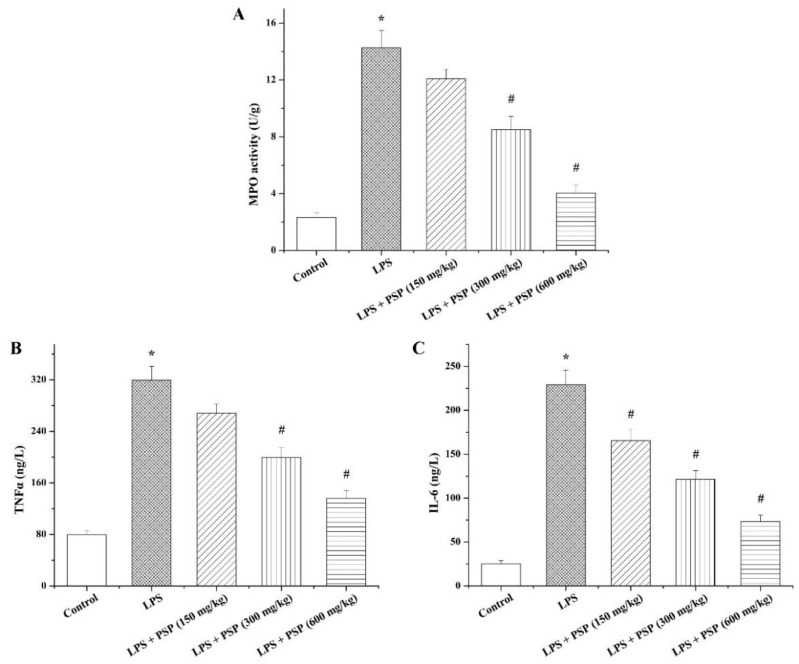
Effects of PSP on the activity of neutrophil infiltration marker MPO in liver tissue and the levels of inflammatory cytokines TNFα and IL-6 in serum of LPS-induced septic mice (*n* = 6). (**A**) Liver MPO; (**B**) serum TNFα; (**C**) serum IL-6. Data are expressed as mean ± SEM. * *p* < 0.05 versus control group, # *p* < 0.05 versus LPS group.

Considering that inflammation has a significant effect on the pathogenesis of sepsis, the activity of MPO, a neutrophil infiltration marker, in the liver was assayed to evaluate the anti-inflammatory effect of PSP (Figure 4A). In keeping with the results of histopathological evaluation, intraperitoneal injection of LPS significantly enhanced the hepatic MPO activity compared with the control group, whereas the elevation of MPO activity caused by LPS was observably abated by PSP in a dose-dependent manner. Moreover, the serum contents of inflammatory cytokines TNFα and IL-6 were tested to further assess the anti-inflammatory action of PSP. As shown in Figure 4B,C, compared to the control group, the levels of TNFα and IL-6 in the serum samples were notably increased in the LPS group, and PSP treatment efficaciously countered the increases in serum TNFα and IL-6 levels induced by LPS.

### 2.4. PSP Ameliorated Hepatocyte Pyroptosis of Septic Mice

**Figure 5:**
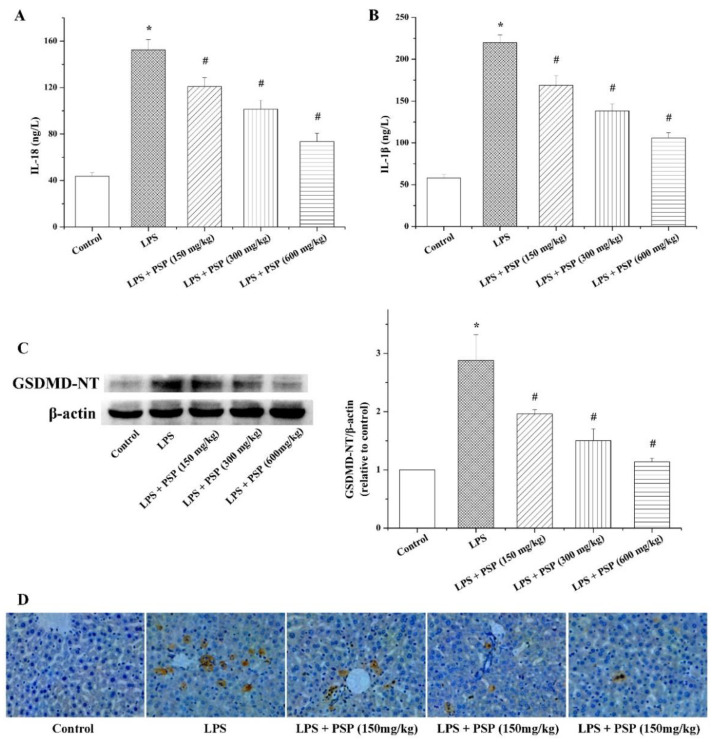
Effects of PSP on hepatocyte pyroptosis of LPS-induced septic mice (*n* = 6). (**A**) Serum IL-18; (**B**) serum IL-1β; (**C**) protein expression levels of GSDMD-NT in liver tissue; (**D**) TUNEL staining images (400×). Data are expressed as mean ± SEM. * *p* < 0.05 versus control group, # *p* < 0.05 versus LPS group.

To illustrate whether PSP restrain ALI via mitigating pyroptosis, serum levels of IL-18 and IL-1β were evaluated. The results showed that the concentrations of IL-18 and IL-1β in serum of the LPS-treated mice were dramatically elevated compared with those of control mice, while PSP administration could distinctly reverse the increase of IL-18 and IL-1β induced by LPS (Figure 5A,B).

To ulteriorly evaluate the impact of PSP on pyroptosis, the protein expression levels of GSDMD-NT, a pyroptosis-executive protein, in liver tissues were tested. The LPS group showed higher levels of GSDMD-NT than that of control group (Figure 5C), and the upregulation of GSDMD-NT protein expression caused by LPS was effectively diminished by PSP treatment. Moreover, the number of TUNEL-positive staining liver cells in LPS-induced septic mice was obviously higher than that in control mice, whereas TUNEL-positive cells were markedly decreased in the PSP treatment group compared to the LPS group (Figure 5D).

### 2.5. PSP Mitigated the Pyroptosis by Inhibiting NLRP3 Inflammasome Signaling

**Figure 6:**
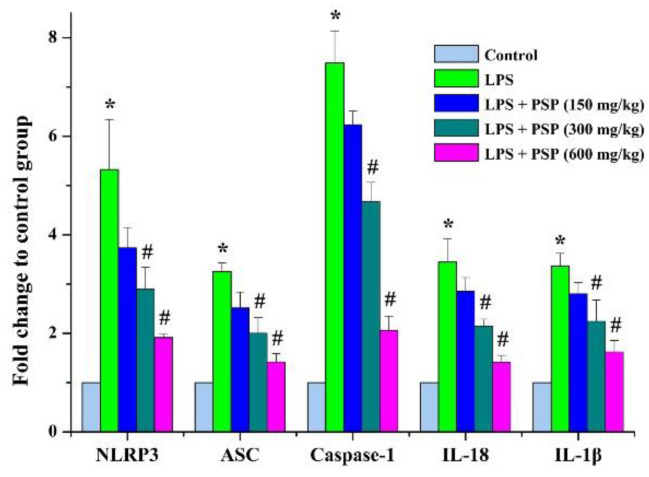
Effects of PSP on mRNA expression levels of NLRP3-inflammasome-signaling-related factors in LPS-induced septic mice (*n* = 6). Data are expressed as mean ± SEM. * *p* < 0.05 versus control group, # *p* < 0.05 versus LPS group.

In order to explore the potential antipyroptosis molecular mechanisms of PSP, the expression levels of factors related to NLRP3-inflammasome-mediated pyroptosis (NLRP3, ASC, caspase-1, IL-1β, and IL-18) were detected using RT-PCR. Compared to the control group, the mRNA expression levels of NLRP3, ASC, caspase-1, IL-1β, and IL-18 were dramatically upregulated after intraperitoneal injection of LPS, while the effects of the upregulations were remarkably suppressed by PSP administration (Figure 6).

## 3. Discussion

Sepsis is an intractable clinical syndrome with high mortality characterized by systemic inflammation and multiorgan dysfunction [32]. Evidence suggests that septic liver injury is an independent predictor of poor prognosis, and repairing liver damage correlates strongly with the decreased mortality of sepsis [10]. PSP have been reported to possess anti-inflammatory and hepatoprotective activities [25]. Therefore, we speculated that PSP might possess a protective effect on septic liver injury. In this study, the sepsis model was established in mice via intraperitoneal injection of LPS and the effects of PSP on SALI were evaluate. The results of this study first indicated that PSP treatment alleviated SALI, and the mechanisms were most likely mediated by attenuating pyroptosis via inhibiting NLRP3/GSDMD signals. Herein, we first explored the effects of PSP on survival rates and liver injury of septic mice. The results of survival analysis demonstrated that PSP treatment markedly reduced the 48 h mortality rate of septic mice. According to the HE staining results, PSP treatment could remarkably alleviate the septic liver injury induced by LPS. Furthermore, the increased levels of liver function indexes AST, ALT, ALP, and TBIL and inflammatory cytokines TNFα and IL-6 in serum caused by LPS were significantly decreased in the PSP treatment group, which was in line with the results of HE staining. In addition, PSP treatment could markedly reduce the activity of neutrophil infiltration marker MPO in liver tissue induced by LPS. All these data indicated that PSP soothed the SALI and therefore lowered the mortality rate of septic mice.

Pyroptosis is a type of cell death depending on inflammatory caspases [33] and takes a significant role in the progression of inflammatory diseases [14]. The marked morphological characteristics of pyroptosis include DNA damage, cell swelling, and lysis caused by pore formation. Eventually, the cellular contents and proinflammatory cytokines (IL-18 and IL-1β) are released, resulting in inflammation [34]. As the substrate of caspase-1/4/5/11, GSDMD is the critical molecule arousing the changes of pyroptotic cells in morphology whose N-terminal domain (GSDMD-NT) oligomerizes in the cell membrane causing pores formation, finally leading to pyroptosis [35]. In order to evaluate whether PSP exerted a protective effect on SALI by inhibiting pyroptosis, the levels of pyroptosis-related inflammatory cytokines IL-18 and IL-1β in serum as well as the protein expression of GSDMD-NT in liver tissue were measured. The results manifested that intraperitoneal injection of LPS dramatically increased the serum levels of IL-1β and IL-18 and the expression of GSDMD-NT. By contrast, these increases were reversed by PSP treatment, which suggested that PSP suppressed excessive pyroptosis and relieved SALI. This speculation was further supported by the results of TUNEL staining. 

Previous research has showed that NLRP3 inflammasome performs important functions in mediating pyroptosis [36]. Assembly of NLRP3 inflammasome could induce activation of caspase-1 and result in the cleavage and excretion of pro-inflammatory cytokines, finally contributing to pyroptosis [37]. Therefore, we detected the mRNA expression levels of the NLRP3/GSDMD signals. The data demonstrated that the upregulations of the mRNA expression levels of NLRP3, ASC, caspase1, IL-1β, and IL-18 were remarkably reversed in PSP treatment groups. Based on these results, we speculated that PSP might exhibit a protective effect against SALI through regulating NLRP3/GSDMD signals.

**Figure 7:**
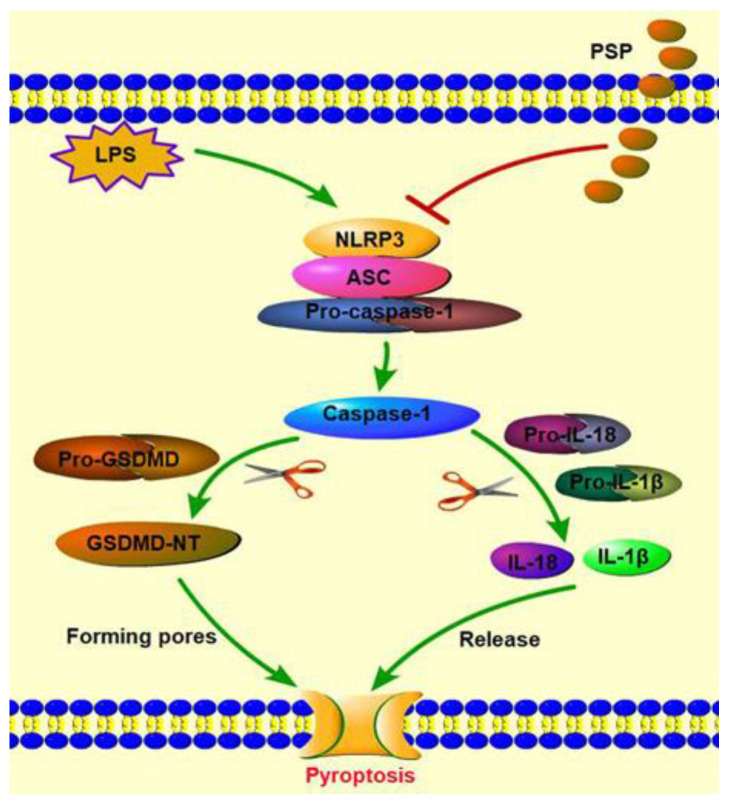
The putative mechanism by which PSP attenuates LPS-induced SALI.

In summary, as shown in Figure 7, our experiments demonstrate a potential protective role of PSP against SALI induced by LPS, which may contribute to inhibiting NLRP3/GSDMD signals. Nevertheless, methodological limitations on this study still remain. The results of the mRNA expression level do not necessarily reflect protein content, and further studies are required to illuminate the underlying mechanism of the protective effect of PSP on septic liver injury.

## 4. Materials and Methods

### 4.1. Animals and Treatments

SPF-grade male BALB/c mice aged 8 weeks were taken from Suzhou Xishan Biotechnology Co., Ltd. (Suzhou, China). All mice were acclimated in a standard laboratory animal room for 7 days before the experiments. All of the experimental procedures conformed to the Animal Experiment Ethics Review Committee of Jiangsu Vocational College of Medicine, Yancheng, China (XMLL-2022-038).

Ninety mice were divided randomly into five groups (*n* = 18): the control group, LPS group, LPS + 150 mg/kg PSP group, LPS + 300 mg/kg PSP group, LPS + 600 mg/kg PSP group. PSP (purity ≥ 98%) used in this study is a commercialized reagent which was purchased from Fufeng Ciyuan Biotechnology Co., Ltd. (Baoji, China). PSP groups were orally administered PSP (150, 300 or 600 mg/kg) once per day for 7 consecutive days. Meanwhile, the control group and the LPS group were given an equal volume of saline. On the seventh day, LPS and PSP groups were intraperitoneally injected with 10 mg/kg LPS (Beyotime, Shanghai, China) after intragastric administration of saline or PSP for one hour, and the control group was intraperitoneally injected with an equal volume of vehicle. After the intraperitoneal injection, animal survival of each group was recorded every 8 h for 2 days. In total, fifty mice (*n* = 10) were used for survival analysis, and another forty mice were used for specimen collection and further analysis. 

### 4.2. Specimen Collection

Twenty-four hours after the intraperitoneal injection of LPS or saline, blood samples were collected into sterile centrifuge tubes by picking the right eyeball of mice and centrifuged to obtain the upper serum. The obtained serum samples were kept in −80 °C until analysis, and the liver tissues were removed and collected after being washed with saline three times. The liver tissues were split into two parts. One part was immersed in 4% paraformaldehyde to fix the tissue for subsequent histological analysis, and another part of the liver tissue was frozen at −80 °C for other detection.

### 4.3. Histological Analysis

After being fixed with 4% paraformaldehyde for 48 h, the liver tissues were dehydrated and embedded in paraffin. Then, the paraffin-embedded liver tissues were cut into 5 μm slices and stained with hematoxylin and eosin (H & E). Thereafter, the stained slices were observed using light microscopy (OLYMPUS, Tokyo, Japan) to assess the histological changes of liver tissues. Summarizing the existing literature, the histological changes of septic liver injury mainly include inflammatory cell infiltration, cell spotty necrosis, and edema. Inflammatory cell infiltration is an inflammatory reaction in which inflammatory cells (neutrophils, lymphocytes, monocytes, and mast cells) in blood vessels or lymphatic vessels pass through the walls and enter the interstitial space. Cell spotty necrosis is characterized by nuclear condensation, hyperchromasia, shrinkage, and even fragmentation or lysis.

### 4.4. TUNEL Assay

The chromatin integrity of the cells in liver tissues were detected using a Colorimetric TUNEL Apoptosis Assay Kit (Beyotime, Shanghai, China). The TUNEL assay was conducted based on the instructions. In brief, after serial processing of dehydration, successive incubation of proteinase K and TUNEL solution, and counterstaining with hematoxylin, the prepared slides were sealed. Finally, the degree of apoptosis was observed using light microscopy.

### 4.5. Biochemical Measurements

To assess the liver damage of mice in each group, the serum levels of aspartate aminotransferase (AST), alkaline phosphatase (ALP), alanine aminotransferase (ALT), and total bilirubin (TBIL) were determined using corresponding commercialized assay kits (Nanjing Jiancheng Bioengineering Institute, Nanjing, China) according to the accompanying instructions. In addition, the concentration detections of inflammatory cytokine TNFα, IL-6 and pyroptosis-related inflammatory cytokines IL-18, IL-1β in the serum were performed using ELISA kits (Beyotime) based on manufacturer’s protocols. Moreover, a MPO assay kit (Nanjing Jiancheng Bioengineering Institute, Nanjing, China) was used to measure the MPO activity in liver homogenate according to the recommendations offered by the manufacturer.

### 4.6. Total RNA Extraction and Quantitative RT-PCR Assay

Extraction of total RNA from liver tissues (up to 30 mg) was conducted using GeneJET RNA Purification Kit (Thermo Fisher Scientific, Waltham, MA, USA) based on the instructions afforded by the manufacturer. OD260/OD280 and OD260/OD230 of the RNA samples were measured using a NanoDrop 2000 spectrophotometer (Thermo Fisher Scientific) to monitor the purity of the samples, and 28S/18S and RNA integrity number (RIN) of the RNA samples were detected with an Agilent 2100 Bioanalyzer (Agilent, USA) to evaluate the integrity of the samples. Then, the extracted total RNA with qualified purity and integrity was programmed to synthesize cDNA using a RevertAid First Strand cDNA Synthesis Kit (Thermo Fisher Scientific) following the manufacturer’s protocols. BeyoFast™ SYBR Green qPCR Mix (Beyotime)-based RT-PCR was performed for quantificational assessment of the specific gene expressions in the StepOnePlus™ Real-Time PCR System (Applied Biosystems, Waltham, MA, USA). The mRNA expressions of specific genes were calculated based on the 2^−ΔΔCt^ method and standardized to β-actin expression as the internal housekeeping control. The primer sequences for amplification of specific gene are listed in Table 1.

### 4.7. Western Blot Assay

Briefly, isolation of total protein from liver tissues was performed using RIPA Lysis Buffer (Beyotime, Shanghai, China) added with Protease-Phosphatase Inhibitor Cocktail (Beyotime, Shanghai, China) on ice, and the supernatants were collected after centrifugation. Protein concentrations were measured using a BCA Protein Assay kit (Beyotime, Shanghai, China). Equal proteins of each group were loaded on 10% SDS-PAGE to separate them and were delivered into polyvinylidene difluoride membrane. The membranes were blocked in 5% skimmed milk, which dissolved in Tris buffered saline with Tween 20. Then, they were incubated with primary antibodies against Cleaved-GSDMD (10,137; 1:1000; CST), β-actin (AF5003; 1:5000; Beyotime) overnight at 4 °C. After being incubated with secondary antibodies (A0208; 1:1000; Beyotime), the blots were visualized with enhanced chemiluminescence BeyoECL Star (Beyotime, Shanghai, China), and the chemiluminescent signals were captured using the ChemiDoC XRS+ system. Finally, the relative intensity of the chemiluminescent signals was quantified using the ImageJ software.

### 4.8. Statistical Analysis

Except for survival rates, which were expressed as percentages, other data were presented as mean ± SEM. The statistical analysis was conducted using SPSS 22.0. One-way ANOVA was used for intergroup comparisons, and a log-rank test was used for comparisons of survival rates. Statistical significance was set as the *p* value less than 0.05.

## 5. Conclusions

Collectively, the present study suggests for the first time that PSP effectively alleviated SALI induced by LPS through inhibiting pyroptosis via regulating NLRP3/GSDMD signals. Thus, our findings demonstrate that PSP might be a potential therapeutic agent for SALI.

## Figures and Tables

**Table 1:** Primers sequence for RT-PCR assay.

Gene	Forward Primer (5′-3′)	Reverse Primer (5′-3′)	Gene ID
IL-1β	TCGCAGCAGCACATCAACAAGAG	TGCTCATGTCCTCATCCTGGAAGG	NM_008361.4
IL-18	ATGCTTTCTGGACTCCTGCC	AGTCTTCTGACATGGCAGCC	NM_008360.2
NLRP3	GAGCTGGACCTCAGTGACAATGC	ACCAATGCGAGATCCTGACAACAC	NM_001359638.1
ASC	GCAACTGCGAGAAGGCTATG	GTGAGCTCCAAGCCATACGA	NM_023258.4
Caspase-1	ACAACCACTCGTACACGTCTTGC	CCAGATCCTCCAGCAGCAACTTC	NM_009807.2
β-actin	CCTAGGCACCAGGGTGTGAT	TCCATGTCGTCCCAGTTGGT	NM_007393.5

## Data Availability

Data used to support the findings of this study are available from the corresponding author upon request.

## Sample Availability

Not available.
